# A Note on Sciatica

**Published:** 1909-12-11

**Authors:** 


### Orthopædics.

### A NOTE ON SCIATICA.

The causes of sciatic pain are so various, and sometimes so important, that it is imperative to examine carefully every case where the patient complains of such pain. While the majority of these causes are well known, there is one, dependent upon an anatomical lesion, which comes more especially under the charge of the orthopædist, and which is by no means so generally recognised as it should be.

Many years ago Billroth pointed out that there were certain cases of sciatica in stout, obese women that presented certain peculiar features. This observation was confirmed by other German and French surgeons, but no attempt was made to identify the variety as a distinct clinical entity, and it remained for Goldthwait of Boston, in 1905, to describe accurately the condition which is now well recognised by orthopædists as sciatica due to weakness of the pelvic ligaments. There is, unfortunately, no distinctive name for this variety. "Subluxation of the sacro-iliac synchondrosis" is not always right, although it is on the whole the most expressive nomenclature that can be adopted. "Goldthwait's sciatica," although it associates a particular writer with the condition, and is therefore likely to create invidious distinctions, is a useful and short enough name, and may therefore be conveniently used, as it will be, in these articles.

### Ætiology and Causation.

This variety of sciatic pain depends primarily on weakness or instability of one or other of the pelvic articulations. Most commonly the main lesion is a subluxation of the sacro-iliac synchondrosis: more rarely the cause is seated in the ilio-lumbar or in the pubic joint, but in every case there appears to be present a definite laxity of the articular ligaments leading to want of apposition of the joint surfaces and a consequent inequality between the two sides of the pelvis. The ætiological factors are not clearly known. The condition is much more frequently met with in women than in men: indeed in its real sense it is unknown in men. There can be no doubt, however, that when pathological or traumatic causes have acted upon the male pelvis similar changes may occur and an identical condition brought about. This is seen in cases of sacro-iliac disease in young males in which sciatic pain is by no means infrequently complained of. For all practical purposes, however, the condition is limited to women, and the chief factor in its causation is undoubtedly repeated pregnancies. Subsidiary factors are inflammatory conditions acting upon the articular ligaments (chief among which are gonorrhœa, syphilis, and rheumatism, though every specific infection may act similarly), trauma, the surgical operations known as hebeotomy, pubiotomy and symphysiotomy, abnormalities and inequalities of the lower lumbar portion of the spine such as spondylolisthesis, and, finally, the acute infective or inflammatory lesions. This last group need not be discussed here: the others are worth a short description. They all act by weakening the tie between the two joint surfaces. Dr. Goldthwait remarks, "As a physiological part of pregnancy the pelvic joints are relaxed, at times to quite an extreme degree, but practically always enough for the character of labour to be greatly modified, while . . . at each menstrual period there is a physiological relaxation of these joints." Usually the resulting weakness is better seen on one side than on the other, but careful examination will often discover evidences of bilateral instability—an important point in the diagnosis. The weakening of the ligaments results, from purely static causes, in a tilting of the pelvis, and this again causes a stretching of or an actual pressure upon the sciatic nerve as it passes beneath the notch, and gives rise to the pain of which the patient complains. Where the pelvic girdle has been weakened by such operations as hebeotomy, the instability is directly due to this: in such cases it may reach an extreme degree. The series of $x$-ray photographs taken at Bumm's clinic at Berlin in pubiotomy cases has clearly shown the bad effects of this operation upon the pelvic articulations. In the cases due to weakening by old inflammatory conditions, the inequality is more commonly seen on one side: in this connection osteo-arthritis must be borne in mind.

### Diagnosis.

In all such cases there are several points which are of the utmost importance in diagnosis. The patients are women, usually multiparæ, and in all cases individuals in whom there is reason to suspect that, through some cause or other, the pelvic articular ligaments have been stretched or weakened. They complain of sciatic pain which is in no way different from the pain which is met with in other varieties of sciatica. There are pain and tenderness along the course of the nerve, and in old cases there are marked wasting and some rigidity of the muscles of the thigh and leg: so far as the nerve is concerned there is a true neuritis due to pressure or stretching. Subjectively there are "feelings of pain," weakness, and "uneasiness" in one or other of the joints of the pelvis: usually a dull ache over the sacrum is complained of. Objectively there are signs of pelvic inequality. In extreme cases the patient may limp or "waddle" with a gait reminiscent of dislocation of the hip. There is usually marked lateral curvature in the dorso-lumbar region. The pelvis itself is tilted, and the anterior superior spines are at unequal levels: when the patient is examined from behind Trendelenburg's sign may be present, as in cases of hip or sacro-iliac disease. In very bad cases there is abnormal mobility of the pelvis, and the joints can be pushed against each other and grating elicited. The pelvic condition influences the whole carriage of the body, and leads to deformities elsewhere, such as the scoliosis and talipes valgus, which may be observed, and which, if the case is left untreated, gradually become worse. Measurements are not of much use. There is no actual shortening in any case except where there has been pathological destruction of bone or a fracture. Such cases of shortening are rarely met with, and the writer has only seen one in which the pelvic inequality resulted in "sciatica" in a young woman who as a child had fractured her pelvis, the line of fracture running obliquely across the acetabulum.

In the differential diagnosis care must be taken not to mistake these signs of pelvic inequality for muscular deformities due to caries of the spine or pelvic bones. Any active disease of the pelvic bones must be excluded, and special care must be taken not to overlook a sacral growth or a deeply-seated aneurysm, in both of which the "sciatica" may be the main symptom complained of. An *x*-ray photograph should be taken wherever possible, as it may give useful information.

### Treatment.

The treatment of this condition is quite simple, and once the diagnosis has been made, there should be no difficulty in easing the patient and rendering her condition more bearable. A permanent cure cannot be looked for. The ligaments cannot be restored to their original strength, and the gaping between the joint surfaces remains. What the orthopædist must do is to prevent this condition of instability becoming worse, and so increasing the patient's suffering. The best way to treat the condition is, therefore, to fix the pelvis artificially, at first by means of a properly applied plaster spica, and later on by means of a well-fitting celluloid hip corset or gutta percha pelvic girdle. No matter what method is adopted, care must be taken that the fixing is at first absolute: there must be no "waggling" allowed, and to obviate this defect it is just as well for the patient to remain in bed for a week or more after the plaster spica has been put on. This relieves the strain on the nerve, which has caused a true interstitial neuritis, and is nearly always successful in relieving the pain as well. Where it fails to ease the patient, the neuritis may be treated locally by counter-irritation or by actual injections into the nerve. It is very rarely necessary to resort to such measures: usually a few days' perfect rest in the recumbent position suffices to relieve the sciatic pain. Then, of course, the pelvis has to be permanently fixed, as already described. For use after the plaster spica, nothing is so efficient as a hip corset made of celluloid and strengthened with light steel stays. This is easily made by the orthopædist who does not disdain to make use of the acetone pot himself; but if there is any difficulty in fitting it on, the corset can be made to measure by an instrument maker. In all cases the medical attendant should satisfy himself that it fits properly and does not inconvenience the patient.

